# The Role of MSC in Wound Healing, Scarring and Regeneration

**DOI:** 10.3390/cells10071729

**Published:** 2021-07-08

**Authors:** Raquel Guillamat-Prats

**Affiliations:** Lung Immunity Laboratory, Germans Trias i Pujol Research Institute (IGTP), 08914 Badalona, Spain; rguillamat@igtp.cat; Tel.: +34-93-554-3050

**Keywords:** wound healing, regeneration, scar, MSC, mesenchymal stem/stromal cells, fibrosis, inflammation, angiogenesis, extracellular matrix

## Abstract

Tissue repair and regeneration after damage is not completely understood, and current therapies to support this process are limited. The wound healing process is associated with cell migration and proliferation, extracellular matrix remodeling, angiogenesis and re-epithelialization. In normal conditions, a wound will lead to healing, resulting in reparation of the tissue. Several risk factors, chronic inflammation, and some diseases lead to a deficient wound closure, producing a scar that can finish with a pathological fibrosis. Mesenchymal stem/stromal cells (MSCs) are widely used for their regenerative capacity and their possible therapeutically potential. Derived products of MSCs, such as exosomes or extravesicles, have shown a therapeutic potential similar to MSCs, and these cell-free products may be interesting in clinics. MSCs or their derivative products have shown paracrine beneficial effects, regulating inflammation, modifying the fibroblast activation and production of collagen and promoting neovascularization and re-epithelialization. This review describes the effects of MSCs and their derived products in each step of the wound repair process. As well, it reviews the pre-clinical and clinical use of MSCs to benefit in skin wound healing in diabetic associated wounds and in pathophysiological fibrosis.

## 1. Introduction

During tissue damage there is a usual response to injury that involves several steps of overlapping events, called wound healing [1]. Wound healing is a dynamic process and many cellular players and structures are involved in the process. These cellular and molecular events are highly coordinated and controlled. For effective tissue repair and restoration of tissue function, there is a need for alteration of actin cytoskeleton as well as secretion of extracellular matrix (ECM) proteins and integrin receptors [2]. Scarring is a way to heal an injury when fibrous tissue replaces the damaged tissue, but on the other side regeneration can happen when the injured tissue is replaced by renewing and rebuilding itself. In humans and other mammals, almost all tissues are prone to heal by forming a scar, and very few tissues are able to regenerate.

The wound repair process has several phases: (1) homeostasis/coagulation, (2) inflammatory cell recruitment, (3) proliferative phase and (4) maturation phase [1,2,3].

**Figure 1:**
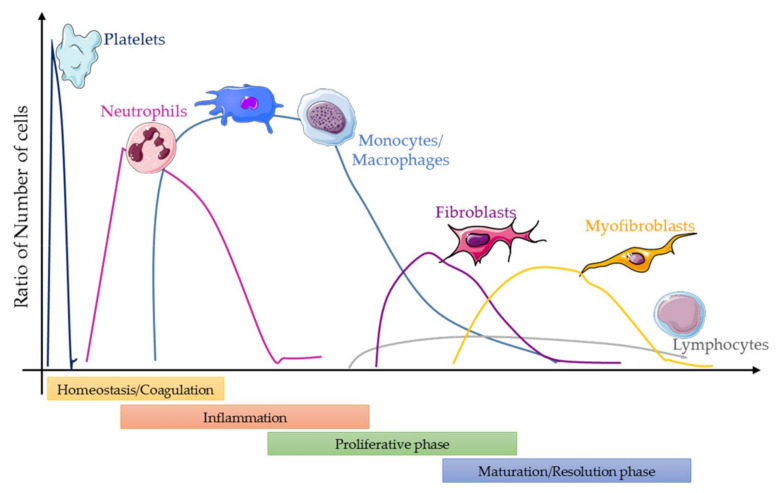
Time lapse of cells recruited into a wound. During the first phase, platelets and neutrophils are recruited, and then monocytes are recruited and infiltrate the tissue and differentiate into macrophages; resident macrophages can also proliferate. Altogether, this leads lead to fibrocyte recruitment and fibroblast proliferation and the posterior conversion to myofibroblasts. Other cells such as lymphocytes—mainly T cells—are also recruited in low numbers at the late stages. This figure does not show the NK, dendritic cells and mast cells; usually their numbers are extremely low, but still they can play an important role on the process.

In the first step, platelets and the activation of the coagulation cascade are the main players, and fibrin strands adhere in the first seconds; there is a formation of thrombus or a clot, and platelets are trapped in the wound area. The inflammatory phase is triggered by the recruitment of inflammatory cells into the wound site, which will try to eliminate the damaged cells, the pathogens of the wound area. The leukocytes recruited into this site—first the neutrophils, then the monocytes, followed by others—secrete growth factors, enzymes and chemokines that produce swelling, heat, redness and pain [4] (Figure 1). If this stage is prolonged, and an excessive number of activated cells are recruited at the injured site, then the inflammation will not help the wound healing process. In the proliferative phase, the main objective is to cover and fill the wound; the margins of the wound start contracting by fibroblasts that are activated and differentiated into myofibroblasts. Afterward, the re-epithelialization process starts; this phase is triggered by extracellular matrix (ECM) deposition, mainly of collagen [5,6,7,8]. Finally, during the maturation phase, the collagen fibers reorganize from collagen type III to type I, and the tissue remodels, slowly gaining strength and flexibility by promoting epithelialization and neovascularization [9,10,11,12].

The failure of the wound healing process is not completely understood, and the abnormal formation of a wound is highly associated with a continuous localized inflammation [13]. There are many risks factors, such as age, malnutrition, infections, smoking, medications or radiation, associated with improper wound healing. Unresolved long-lasting inflammation favors scarring over regeneration, and the control of the inflammatory response at early stages may be critical for regeneration [4,14,15]; as mentioned previously, in mammals, scarring is the standard process to resolve tissue damage. Resolving inflammation is connected not only to the innate immune system, the adaptive immune system also plays an important role in the process [16]. The regenerative process is highly regulated by the expression of several proteins and chemoattractants, the recruitment of several immune cells at different time points and in diverse cell numbers, and the activation of the regenerative and angiogenic pathways [17,18] (Figure 1).

ECM remodeling is essential for proper wound healing, and the regulation of activity of the matrix metalloproteinases (MMPs) and tissue inhibitors (TIMPs) is meticulously controlled [5,6]. Fibroblasts and myofibroblasts secrete MMPs, enzymes involved in remodeling of type III collagen to type I collagen to close the wound, among other important conversions, and this may change the strength of the healing tissues. Excessive amounts of collagen synthesis or abnormal collagen turnover and exaggerated ECM can accumulate in these wounds, causing a scar [5,6]. Keloids and hypertrophic scars are fibroproliferative malignant processes characterized by excess accumulation of collagen and other ECM proteins. In these disorders, there is an abnormal inflammation and excessive secretion of pro-fibrotic cytokines and ECM together with abnormal cell proliferation and migration [19,20,21].

Diverse organisms repair their tissues and organs in different ways; numerous organisms are able to regenerate damaged tissues and organs completely. However, other organisms replace the injured tissue with pathological connective tissue, called a scar [22]. In humans, perfect tissue regeneration is not produced, and the tissue is repaired by excessive ECM, leading to tissue fibrosis. Many organs are affected by scarring after damage, producing chronic diseases and loss of function of the organ, such as liver fibrosis or pulmonary fibrosis. Additionally, it is well known that hypertrophic scars can revert, but usually fibrosis in soft tissues does not regressing and might lead to organ failure.

There are many pathologies that can affect the wound healing and scarring processes, such as diabetes, obesity, hypertension and vascular diseases [23]. Many wounds need intensive treatment, such as necrotic wounds, ulcers, diabetic wounds, extremity wounds with edema and chronic wounds [24].

Many studies have looked for new developments to manage one of the steps of the reparation and regeneration process [25]. Drugs with antibacterial properties, attracting immune cells, triggering a proper ECM formation, and growth factors have been used as therapeutical efforts to improve tissue regeneration. Platelet-derived growth factor (PDGF), fibroblast growth factor (FGF-2), keratinocyte growth factor (KGF-1), vascular endothelial growth factor (VEGF), granulocyte-macrophage colony-stimulating factor (GM-CSF) and granulocyte colony-stimulating factor (G-CSF) have been used as possible therapies for regulating wound healing [26,27,28,29,30,31,32,33,34,35].

In the last years, cell therapy treatments have entered the playing field as possible therapies. Mesenchymal stem/stromal cells (MSCs) are multipotent stromal cells that can differentiate into mesenchymal tissue lineages such as osteoblasts, chondrocytes, myocytes and adipocytes, but not to hematopoietic stem cells. MSCs are found in most tissues, mainly in bone marrow and adipose tissue; however, their diversity and lineage connections are not yet totally understood. MSCs have been used in disease models to control several steps of the wound healing and regeneration process [36,37]. MSCs have been used to treat cancer, diabetes, neurological disorders, cardiovascular and pulmonary diseases and many more. Overall the positive effect of MSCs in the process seems to be a paracrine-derived effect [36,38,39,40]. Depending on the pathology to be treated, the administration route of the MSC is also a topic that needs to be taken into account; sometimes a local administration will reduce the side effects and extend and increase the therapeutic effect. The Food and Drug Administration (FDA) in the US has approved several cellular products for regenerative purposes to be used on the clinic (specifically regulating wound care products containing live cells), and the European Medicines Agency (EMA) also has made an effort to regulate the use of cell-based therapies in the last years [41]. The advances in stem cell biology have improved the noticeable limitations of the use of primary cells and cell-derived therapies.

**Figure 2:**
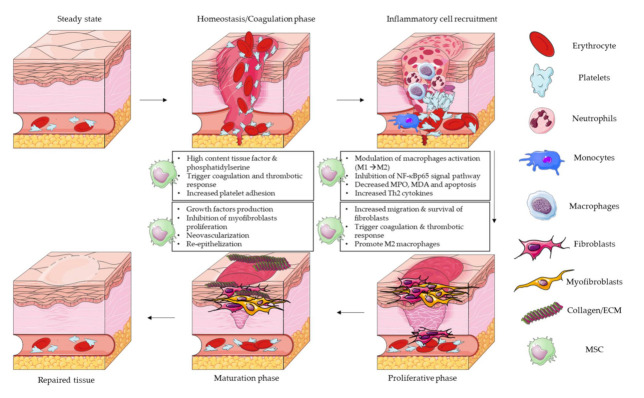
Wound healing process and role of mesenchymal stem/stromal cells in each step of the process. The illustration shows the cellular players in each phase and summarize the main functions of MSCs in each step. (MSC: mesenchymal stem cell; ECM: extracellular matrix; MPO: myeloperoxidase; MDA: malondialdehyde).

In this review, we assess the properties and main functions of MSCs in each step of the process of wound healing and why they can be useful and interesting as therapeutic treatment. We focus here on pathologies and injuries that are driven by a fibroproliferative process. MSCs per se are not the only elements used in these studies; MSC-derived products such as exosomes or extracellular vesicles (EVs) have shown to present a therapeutic effect for wound healing and the regeneration of tissue. All of the described effects are related to MSCs or MSC-derived products (Figure 2).

## 2. MSC Function in Each Step of the Wound Repair Process

### 2.1. MSCs Regulating Homeostasis Phase

Several studies have described that MSCs promote coagulation due to the high content of phosphatidylserine and tissue factor (TF) on their surface. Moreover, EVs derived from MSC-conditioned medium also contain TF and phosphatidylserine on the surface, which also triggers coagulation. The expression of these two factors triggers a thrombotic response, which can increase the formation of clots [42]. This is one of the main complications associated with the administration of MSCs for several diseases: the risk of therapy-induced thrombosis [43,44,45,46,47,48,49]. Other studies described the same effect, noting the presence of Annexin V on the MSC surface, implying the presence of phosphatidylserine, which triggers clot formation [50] (Figure 2).

Chance et al. claimed that EVs interfere with platelet adhesion in an in vitro assay [42]. However, the addition of MSCs or their derived EVs to platelet-free-plasma triggers the formation of fibrin clots, suggesting that platelets are not needed to induce clots by the MSC.

In in vivo models, the use of adipose-derived MSCs presented a procoagulant effect during endotoxemia [51]. Several studies revealed no side effects or animal deaths from thrombosis during administration of MSCs. On the other hand, the described rare clinical cases of thromboembolism [8,9,10,11] associated with MSC transplantation require more detailed analysis [43,45,46].

### 2.2. MSCs Modifying Inflammatory Phase

The inflammatory phase is one of the main steps for deciding a normal or impaired wound healing course; this phase is necessary to clean bacteria, tissue debris, apoptotic cells and clots from the wound. Habitually, systemically administered MSCs migrate to sites of damage and can interact with leukocytes to regulate their response. MSCs secrete several growth factors and cytokines that can regulate the response of neutrophils, macrophages and lymphocytes. Numerous mechanisms, using different animal models, have been suggested to explain how MSCs improve tissue regeneration using their anti-inflammatory properties [52,53] (Figure 2).

For example, it has been shown that MSC secretome is able to modulate macrophage response; Zhang et al. showed that during skin wound healing, MSCs were able to polarize macrophages from a pro-inflammatory M1 to reparative/anti-inflammatory M2 activation [54,55]. In addition, and supporting the previously mentioned studies, Jian et al. showed that MSCs are able to suppress the pro-inflammatory TNF-α released from M1 macrophages and increase the TGF-β1-dependent induction of myofibroblast-driven wound contraction [56]. As well, MSCs control Th1-Th2 cytokine balance, triggering the production of anti-inflammatory cytokines such as IL4, decreasing the production of the pro-inflammatory IFNy and having a suppressor effect on NK activity and cytotoxicity [36,57]. The switch of activation of the macrophages from an M1-inflammatory phenotype to a M2-reparative/anti-inflammatory one is a key step for wound healing and to control inflammation.

EVs derived from bone marrow MSCs were able, per se, to down-regulate pro-inflammatory cytokine expression, inhibit NF-κBp65 signal transduction pathway and balance anti-oxidant/oxidant compounds in an experimental model of colitis. EVs derived from MSCs showed a proper therapeutic effect in a rat colitis model by decreasing myeloperoxidase (MPO) activity, malondialdehyde (MDA) and apoptosis (caspase-3, caspase-8 and caspase-9) [58].

Evidence has shown that MSC therapy is likely to reduce inflammation, for example, in acute and chronic liver injury [59]. The use of MSCs is a strategy widely used in the last years to battle organ inflammation in different compartments [60]. For SARS-CoV-2 infection, MSCs and some derivative products were suggested as a treatment for lung and liver infection [61,62,63].

### 2.3. MSCs Improving Proliferative Phase

In this phase, the main objective is to cover and fill the wound in a proper way. There is a need for the margins of the wound to start contracting by fibroblasts/myofibroblasts; however, in parallel, there must be a proliferation and recovery of the epithelial cells. During this step, there is a main regulation of collagen, the production of other ECM proteins and a balance of the ratio between fibroblasts and myofibroblasts. During the proliferative phase, the main key players are the fibroblasts, but macrophages or T cells can also modulate their activation. In addition, the most important aspect is the start of the re-epithelialization procedure, which is the limiting element to regenerate the tissue (Figure 2).

The treatment with MSCs enhances survival and migration of fibroblasts and increases the ECM deposition by fibroblasts, enhancing the healing effects [64]. Derived products of MSCs, such as exosomes, also led to collagen deposition and played and antifibrotic role in hypertrophic scars [65]. Exosomes also facilitated the proliferation and migration of fibroblasts [66].

To promote the wound healing step, MSCs modulate the production of effector T-cell cytokines and polarize the macrophages to a M2-reparative/anti-inflammatory activation, leading to tissue repair [67,68].

Finally, extravesicles derived from MSCs have promoted epithelial cell proliferation in cutaneous wound healing in a rat skin burn model. In vivo, MSCs increased the expression of CK19, PCNA and collagen I (compared to collagen III) [69]. The regeneration of dermal tissue was also promoted by MSCs obtained from adipose tissue [70]. Mesenchymal stem co-cultured with fibroblasts induced dermal fibroblast responses to injury, accelerating fibroblast migration [71].

Zhang et al. modified MSCs to increase their efficiency to differentiate to epithelial cells and improve the re-epithelialization of the alveolar epithelium in lipopolysaccharide (LPS)-induced acute respiratory distress syndrome (ARDS) in a mice model [72]. Several studies have confirmed that MSC transplantation into the lung was able to reduce lung damage in acute lung injury mice models, suggesting a role for MSCs in improving lung alveolar epithelial cell proliferation and alveolar epithelium regeneration [73,74].

Scleroderma is an autoimmune disease that produces mainly general skin fibrosis but also can develop into several organs. MSC-based therapy is able to counteract the multi-visceral fibrosis shown in this systemic pathology, and an injection of MSCs allowed investigators to limit the pro-inflammatory and pro-fibrotic bleomycin systemic effect through a mechanism involving IL-1RA [75,76,77]. In several preclinical studies using a scleroderma model, MSCs decreased skin thickness, the expression of Col1, Col3 and α-Sma transcripts, and collagen content in skin and lungs [78,79]. The anti-fibrotic effect was associated with a reduction of TNFα and IL1β as well as an increased ratio of Mmp1/Timp1 [78].

### 2.4. MSCs Amending Maturation Phase

The maturation phase is the last step of the process of wound repair, and during this phase, collagen fibers must reorganize properly and tissue must remodel, slowly gaining strength and flexibility. During this step, together with the previous one, it will be determined if, finally, the tissue is left with a scar or really regenerates (Figure 2).

It is well known that MSCs release numerous cytokines and growth factors with anti-fibrotic properties, for example, the hepatocyte growth factor (HGF), IL-10 and adrenomedullin [38,55,80]. MSCs that migrate into the wound secrete HGF and PGE2, and both of these factors are able to inhibit the myofibroblast differentiation and avoid the epithelial-mesenchymal transition [81,82].

MSC signaling triggers other neighbor cells to produce the correct ECM, resembling the correct dermal tissue [71] and also secreting several factors that promote vascular stability and vasoprotection [83,84].

One of the main functions at this stage of wound repair is to improve the vascular formation [85] and to enable the development of functional vasculature [86,87]. Without neovascularization, which includes vasculogenesis and angiogenesis, the acute wounds will become chronic wounds [88], and EVs derived from several sources of MSCs have been shown to stimulate an angiogenic response in vivo [89,90,91,92]. MSCs may contribute to neovascularization in adults by the release of proangiogenic factors such as HIF-1, VEGF, EGF and CXCL12.

## 3. MSCs as a Treatment of Several Typical Pathologies Regarding Wound Healing

### 3.1. MSCs for Treating Skin Wound Healing

The skin provides us a protective barrier against physical damage and infections and maintains body homeostasis [93]. After skin damage, there is an activation of several mechanisms to restore it. Skin wound healing is a multifaceted process that connects cell proliferation and migration together with the production of ECM; leukocytes, resident cells, ECM, chemokines and several growth factors participate in the process [94]. Aging skin is linked to the impossibility of a proper healing [95].

In clinical studies, the local application of cultured autologous MSCs to the wound using a fibrin polymer spray is able to augment the repair process in patients with chronic, long-standing, non-healing lower extremity wounds [96]. The local and sustained effect of MSCs applied with a matrix or hydrogel to the wound directly enhances the reparative and therapeutic effect of the MSCs. Dash et al. showed that autologous cultured bone-marrow-derived MSCs accelerated the healing process and improved the clinical parameters significantly in 24 patients with non-healing ulcers [97].

When bone-marrow-derived MSCs were cultured in a hydrogel and applied to a skin wound, the treatment promoted wound closure, epithelial cell proliferation and re-epithelialization and reduced inflammation in severe skin lesions in a mouse model [98].

MSCs have beneficial effects on the wound healing process and accelerate skin wound healing, collagen deposition, neovascularization and cellular infiltration, improving skin injuries [99]. Intraperitoneal and local administration of MSCs promoted skin wound healing by releasing the growth factor VEGF and decreasing the amounts of pro-inflammatory cytokines in a preclinical study [100]. In addition, Sasaki et al. injected MSCs intravenously in mice subjected to several skin wounds, and they suggested that MSCs contributed to skin tissue repair by differentiating themselves to cell components of the skin [101].

The capacity of reparation of skin damage by MSCs was also associated with their capacity to down-regulate TNF-α-dependent inflammation, triggering TGF-β1 production and increasing the number of M2 macrophages, which led to a myofibroblast [20,56,102].

Exosomes derived from human MSCs inhibited dermal fibroblast-myofibroblast transition, inhibiting the TGF-β1/SMAD pathway [103].

Skin wounds treated with adipose-tissue-derived stem cell spheroids triggered wound closure and promoted angiogenesis [104]. In other studies, adipose-tissue-derived stem cells have shown the capacity to activate collagen and elastin deposition by fibroblasts and reduce scarring, preventing myofibroblast formation [58,91]. Bura et al. performed a phase I trial with seven patients using adipose-tissue-derived MSCs to treat chronic ulcers and ischemic limb injuries, and they showed that MSCs improved wound healing by reducing leg pain, ulcer size and pain-free walking distance [105].

The injuries produced by severe burns are extremely challenging problems in clinics, and the use of MSCs combined with biomaterials and gels showed a therapeutic potential, minimizing damage and improving the coverage of the wounds [106]. The clinical utility of MSCs to improve burn wound healing is based on repairing cellular substrates, attenuation of inflammation, and enhancing migration of reparative cells and angiogenesis [107]. Experimental studies have shown the therapeutic effect of MSCs in healing burn damage by promoting angiogenesis [108]. When locally added to a film and matrix, MSCs can reduce inflammation, promote cell repair and improve grafting [106,109]. In humans, extensive skin burns were treated with MSCs, leading to more rapid healing of donor zones, promoted neoangiogenesis and accelerated rehabilitation of the patients by reducing hospitalization length [110,111].

### 3.2. MSCs for Handling Diabetic Wounds

Diabetic wounds involve several pathological processes in patients with diabetes due to hyperglycemia and the blockage of peripheral blood vessels, producing a wound or ulcer (diabetic foot ulcers). MSCs play a positive effect in diabetic chronic and ischemic wounds. Several preclinical studies in rats have shown the efficiency of using MSCs to treat diabetic foot ulcers [112,113]. Inflammatory leukocytes-neutrophils and macrophages contribute to postponed healing in chronic ulcers. The excessive amount of pro-inflammatory cytokines delays healing and promotes chronic inflammation.

Chronic wounds in diabetic feet were also treated with MSCs with positive effects, increasing healing [114]. Local and systemic administration of MSCs into wound models of type 2 diabetes mellitus (T2DM) rats showed an accelerated wound healing by increasing angiogenesis, which was able to increase tissue regeneration [115]. This study showed that treatment with MSCs accelerated wound closure, improved granulation, triggered angiogenesis (mainly increasing VEGF) and facilitated re-epithelialization. The administered MSCs secreted several bioactive factors, able to recruit other cells to repair the tissue in rodents [56,116]. The secretion of trophic factors that improve the wound healing is the main hypothesis for the therapeutic effect of the MSCs [117]. Other studies observed the same effects and described an increased epithelialization, granulation tissue formation and capillary formation in a diabetic mouse model [118]. Topical application of autologous MSCs also stimulated closure of full-thickness wounds in diabetic mice (db/db) [96].

Chronic inflammation, one of the hallmarks for scarring and chronic wounds, is the impairment of macrophages to switch and modulate their activation. The accumulation of M1-pro-inflammatory macrophages correlates with chronic diabetic wounds; MSCs are able to modulate human and mouse macrophage activation, reducing M1 activation and promoting an anti-inflammatory response [119].

BM MSCs have shown partial efficiency as a therapy for diabetic wounds in non-diabetic and diabetic mice by triggering re-epithelialization and angiogenesis and promoting leukocyte infiltration [108,120]. Following the same idea, Chen et al. also showed that exosomes derived from MSCs are able to trigger angiogenesis, facilitating diabetic wound repair [121]. Neovascularization was improved by MSCs in chronic wounds in diabetic rats, promoting a quicker wound closure [122].

### 3.3. MSCs as Treatment for Organ Fibrosis

The complete tissue regeneration of damaged tissues and organs does not usually happen in humans, and under normal conditions, the damaged tissue is replaced by connective tissue; this scarring process results in a non-reversible fibrosis and produces the loss of functionality in several organs. Tissue remodeling by fibroproliferation is an extremely conserved protective response to tissue damage. Several treatments are used to treat fibrosis, and MSCs have shown to have some therapeutic effect.

MSCs and exosomes derived from MSCs have been shown to ameliorate cardiac, renal, liver and pulmonary fibrosis [123]. It has been reported that exosomes also inhibit the bioactivity of keloid fibroblasts [124,125,126,127].

Hepatic fibrosis is considered as a wound-healing response to liver injury. Ultimately, an excess of fibrosis can end in cirrhosis and liver failure. It has been described that the exosome-enriched miRNAs play a role in the pathogenesis of visceral fibrosis and tissue regeneration [128,129,130,131]. In preclinical animal models, MSCs isolated from adipose tissue were shown to alleviate the progress of fibrotic diseases [132,133,134]. Li et al. described that human exosomes derived from MSCs regulate the expression of collagen and p-Smad2, alleviating liver fibrosis in vivo [135]. Same-pathway SMAD was described to be modified by the exosomes secreted by MSCs in an injured endometrium repaired by fibrosis [136]. Takeuchi et al. analyzed the proteome of EVs derived from MSCs, describing an increase in anti-inflammatory macrophage-inducible proteins (e.g., annexin-A1, lactotransferrin and aminopeptidase N), which triggered liver regeneration after fibrosis and cirrhosis; they pre-conditioned the EVs with an IFN-γ treatment, and the pre-conditioning-altered sEVs resulted in efficient tissue repair, indicating a new therapeutic strategy to treat fibrosis in a mouse model of cirrhosis [137].

In heart disease, several studies using MSCs and their derivates have been published. It has been proposed that MSC-derived exosomes are effective for reducing myocardial ischemia and reperfusion damage [135,138]. Hu et al. described the proangiogenic effects on endothelial cells in vitro, stating that exosomes derived from human amniotic fluid MSCs alleviate cardiac fibrosis via enhancing angiogenesis [139].

MSCs offer a treatment for peritoneal fibrosis; serum-free culture conditions enhanced the antifibrotic abilities of MSCs by suppressing inflammation, and their administration may be a potential therapy for preventing peritoneal fibrotic progression [140]. In addition, in kidneys, MSCs have been shown to promote M2 macrophage polarization and attenuate renal fibrosis via transferring HGF in rats [141].

Systemic sclerosis (SSc) is a potentially lethal and rare disease affecting all connective tissues and producing a diffuse fibrosis. MSCs and their secreted EVs have proven efficacy in slowing down the course of the disease. Rozier et al. described an improvement in skin and lungs [76].

To complete this section, we focus in lung fibrosis. Many preclinical and clinical studies using MSCs and their secreted compounds have been performed during the last decade for the treatment of interstitial lung diseases [142,143,144]. Several studies describe the anti-inflammatory, microbicidal, angiogenic and antifibrotic effects of MSCs in preclinical studies of pulmonary fibrosis, and the treatment with these cells was able to improve lung function and reduce mortality in rodent models of chronic lung diseases [145,146,147,148]. After MSC administration into the lung, cells were rapidly removed, and their therapeutic effect seems to be linked to their secretome [149]. For example, a reduction has been shown in the expression of pro-inflammatory (IL-1b, TNF-β, etc.) and pro-fibrotic (bFGF, CTGF, etc.) transcripts in fibrotic lungs treated with MSCs in mice [150]. In addition, it has been described that MSCs promote epithelial cell wound repair in an in vitro scratching assay with A549 cells [151]. Gad et al. showed in a rat fibrosis lung model induced by bleomycin that MSCs ameliorate lung fibrosis by reducing the expression of TGF-β/SMAD pathways [152]. In EVs produced by human bone marrow, MSCs modulated lung macrophage phenotypes and reduced the proportion of proinflammatory alveolar macrophages and classical monocytes in a bleomycin-induced pulmonary fibrosis model [153].

## 4. Conclusions

Wound healing represents an important medical problem. Fibrosis and tissue regeneration are opposite processes related with wound repair. Many factors in the wound healing process are still not well understood, but the role of the MSCs in the process seems to be useful. New studies need to be performed to find the best administration route of the MSC; in many pathologies, a local application will reduce side effects compared to intravenous administration. More in-depth study of the pathways that MSCs or their derivative products activate at the different phases of the disease and how to regulate them in a more efficient way will be a challenge for scientists in the future. Hence, to study the signaling pathways involving MSCs and inflammation, ECM deposition, angiogenesis and epithelialization are necessary to understand how to prevent the formation of scars and to balance scarring with regeneration without loss organ functionality.
